# Brief Summary of Potential SARS-CoV-2 Prophylactic and Treatment Drugs in the Emergency Department

**DOI:** 10.5811/westjem.2020.3.47328

**Published:** 2020-03-31

**Authors:** Cortlyn Brown, Jeanne Noble, Zlatan Coralic

**Affiliations:** * University of California San Francisco, Department of Emergency Medicine, San Francisco, California; † University of California San Francisco School of Pharmacy, San Francisco, California

## Abstract

As of March 30^th^, 2020 there were 161,807 total cases and 2,953 total deaths of SARS-CoV-2 in the United States, with the number of cases expected to rise. Other than supportive care, there are no SARS-CoV-2 specific treatments available for patients discharged from the emergency department (ED) or those admitted to the hospital. In addition, there are no vaccines available to protect our at-risk healthcare workers. The National Institutes of Health is conducting a Phase 1 clinical trial to evaluate for a potential vaccine and the recipients have started to receive the investigational vaccine.^2^ We present a brief overview of the potential prophylactic and treatment agents under investigation, some which could be initiated in the ED if proven effective.

## INTRODUCTION

As of March 30^th^, 2020 there were 161,807 total cases and 2,953 total deaths of SARS-CoV-2 in the United States,^1^ with the number of cases expected to rise. Other than supportive care, there are no SARS-CoV-2 specific treatments available for patients discharged from the emergency department (ED) or those admitted to the hospital. In addition, there are no vaccines available to protect our at-risk healthcare workers. The National Institutes of Health is conducting a Phase 1 clinical trial to evaluate for a potential vaccine.^2^ We present a brief overview of the potential prophylactic and treatment agents under investigation, some of which could be initiated in the ED if proven effective.

## METHODS

We conducted a literature search on March 17^th^, 2020 (updated March 30^th^, 2020) on PubMed and the Cochrane Library. Search terms included “COVID-19/SARS-CoV-2 treatment or prophylaxis or drugs or therapy.” Abstracts of relevant papers were reviewed by the authors. Drug information was obtained from Lexicomp Online.^3^

## CONVALESCENT SERUM

Human convalescent sera has been used since the early twentieth century and is currently under investigation for prophylaxis and treatment.^4^ To obtain the serum, blood is drawn from a subject who has recently recovered from the target disease. After processing, blood that is noted to have high titers of neutralizing antibodies is then administered to high risk individuals such as those with underlying medical conditions, healthcare providers, or those with confirmed exposure.

Convalescent sera has also been used in modern times. It was used during the H1N1 influenza outbreak and found to reduce respiratory viral burden, cytokine response, and mortality in H1N1 infected ICU patients.^5^ Convalescent sera has also been used for other coronaviruses. Eighty SARS-CoV positive patients treated with convalescent serum before day 14 of illness had improved prognosis compared to those treated later.^6^ There are case reports of convalescent sera administration during the current SARS-CoV-2 outbreak in China, but few details are available.^7^ In addition, the drug maker Takeda is working on developing a SARS-CoV-2 immunoglobulin serum.^4^

To enter a cell, coronaviruses depend on the binding of the viral spike proteins to cellular receptors as well as S protein priming by host cell proteases. Hoffmann *et al* found that SARS-CoV-2 uses the SARS-CoV angiotensin converting enzyme 2 (ACE2) receptor to enter cells and the serine protease TMPRSS2 for S protein priming.^8^ Hoffmann *et al* utilized this information and found that convalescent SARS-CoV serum, which is known to have a neutralizing antibody against the viral S protein, decreased SARS-CoV-2 entry into the cell *in vitro*. They also found that rabbit serum raised against the S1 subunit of the SARS-CoV virus inhibited SARS-CoV and SARS-CoV-2 entry into the cell *in vitro*. These data suggest that neutralizing antibody responses raised against SARS-CoV might offer protection against SARS-CoV-2. In addition, given that TMPRSS2 is required for entry, a TMPRSS2 inhibitor, although not convalescent serum, is another possible target.

There are, however, known risks of convalescent sera including possible allergic reaction to other products in the sera and transmission of other diseases. There are also theoretical risks, most notably antibody-dependent enhancement of infection as well as the possibility that antibody exposure may attenuate the immune system leaving individuals vulnerable to subsequent reinfection.^9^

The feasibility of extracting, processing, and providing the serum to ED clinicians for use is a challenge. A commercially available product could be easier to procure and administer. However, at this time there is no formal recommendation or guidelines to initiate this therapy for patients in the ED or those clinicians working directly with COVID-19 patients.

## OTHER POSSIBLE PROPHYLACTIC OR TREATMENT AGENTS

SARS-CoV, MERS-CoV, and SARS-CoV-2 are all betacoronaviruses. Although no prophylaxis or treatment exists against SARS-CoV-2, there are several drugs which have been subject to limited trials or are currently under investigation.^10^ These drugs include remdesivir, chloroquine, hydroxychloroquine, azithromycin, lopinavir-ritonavir, tocilizumab, sarilumab, and losartan.

Both remdesivir and chloroquine have shown some efficacy against SARS-CoV-2. Remdesivir incorporates into nascent viral RNA chains causing pre-mature termination whereas chloroquine increases the endosomal pH required for viral/cell fusion and interferes with glycosylation of cellular receptors.

Wang *et al* showed that remdesivir and chloroquine had a high selectivity against SARS-CoV-2 and blocked viral infection *in vitro* in African green monkey cells.^11^ Remdesivir interfered after the virus had entered the cell, whereas chloroquine interfered at entry and post-entry stages. More importantly, their preliminary data suggest the same effects in human cell lines.

In the U.S., remdesivir is currently only available as an investigational agent or through compassionate use protocols for confirmed SARS-CoV-2 hospitalized patients with invasive mechanical ventilation.^12^ At this time, the use of this agent in the ED is unlikely.

**Population Health Research Capsule**

What do we already know about this issue?

There are now over 150,000 individuals, worldwide, with COVID-19, the disease caused by the SARS-CoV-2 virus.

What was the research question?

This paper summarizes the literature on potential prophylactic and treatment drugs in consideration for COVID-19.

What was the major finding of the study?

There is limited data supporting all potential COVID-19 treatment and prophylactic drugs but some show promise.

How does this improve population health?

A potential treatment or prophylactic drug for COVID-19 could halt the course of the current pandemic and save lives.

Chloroquine and hydroxychloroquine are oral medications that could potentially be used to treat outpatients and those admitted to the hospital. However, as with this author’s institution, these agents may be restricted for COVID-19 indications in the ED until further evidence is available with several exceptions including very sick patients that are admitted. As these agents are available in the community, ED clinicians should exercise caution counseling patients inquiring about chloroquine and hydroxychloroquine, especially with the recent report of a fatal chloroquine overdose intended for COVID-19 self-treatment.^13^

There are currently over 10 clinical trials in China evaluating the effect and safety of chloroquine in the treatment of SARS-CoV-2 associated pneumonia. Although preliminary data have not been released, Gao *et al* reported in a news briefing by the State Council of China that chloroquine phosphate had “demonstrated marked efficacy and acceptable safety in treating COVID-19 associated pneumonia in multicenter clinical trials.”^14^,^15^

Hydroxychloroquine is a chloroquine analog with fewer drug-drug interactions. Yao *et al* studied African green monkey cells *in vitro* and found that chloroquine and hydroxychloroquine decreased SARS-CoV-2 viral replication in a concentration-dependent manner but that hydroxychloroquine was more potent.^16^ They also evaluated physiologically-based pharmacokinetic models *in vivo*. Based on these results, they recommend an oral loading dose of 400 mg hydroxychloroquine sulfate twice daily on day one followed by a maintenance dose of 200 mg twice daily for four days.

Jun *et al* conducted a prospective study of 30 SARS-CoV-2 positive patients randomized to either standard treatment or hydroxychloroquine. They found no difference between the two groups with respect to median duration from hospitalization to undetectable serum SARS-CoV-2, median time to body temperature normalization, and development of diarrhea and liver function test abnormalities.^17^ Gautret *et al*, however, found that the addition of azithromycin to hydroxychloroquine improved virus elimination.^18^ There are current studies investigating the use of hydroxychloroquine chemoprophylaxis for healthcare workers (ClinicalTrials.gov: NCT04318015).

Cao et al conducted a randomized, controlled, open label trial of lopinavir-ritonavir with 199 SARS-CoV-2 hospitalized adult patients with an oxygen saturation less than or equal to 94% on room air or partial pressure of oxygen to fraction of inspired oxygen less than 300 This cohort would then include mild, moderate as well as severely hypoxic patients.^19^ They found no difference in 28 day mortality in the lopinavir-ritonavir group compared to the standard-care group (19.2% vs 25.0%; 95% confidence interval [CI] −17.3 to 5.7) or percentages of patients with detectable viral RNA at various time points. They also noted significant gastrointestinal side effects and stopped the lopinavir-ritonavir early in 13 patients (13.8%) because of adverse events. The lack of survival benefit as well as the significant side effects make this drug unlikely to be used in the ED at this point.

There are also ongoing studies with monoclonal antibodies tocilizumab (ClinicalTrials.gov: NCT04317092) and sarilumab (ClinicalTrials.gov: NCT04315298). Both of these agents are interleukin −6 receptor antagonists that could theoretically attenuate cytokine and acute phase reactants.^3^ Further, as the SARS-CoV-2 depends on the ACE2 receptor for entry, another multicenter placebo-controlled trial is enrolling patients evaluating losartan (angiotensin 2 receptor blocker) in patients requiring hospitalizations (ClinicalTrials.gov: NCT04312009).

## SUMMARY

Convalescent sera has been used in the past as viral prophylaxis and treatment but there is currently a lack of human data on use against SARS-CoV-2. Remdesivir and chloroquine (or hydroxychloroquine) are two medications that have also shown promising results *in vitro* but clinical trial data have yet to be released. Press releases on these medications have discussed promising results. At this time there is no evidence or consensus to initiate these therapies in the ED.
